# Aligning the planets: The role of nurses in the care of patients with non‐ST elevation myocardial infarction

**DOI:** 10.1002/nop2.69

**Published:** 2016-11-04

**Authors:** Christi Deaton, Rachel Johnson, Maggie Evans, Adam Timmis, Justin Zaman, Harry Hemingway, Jacqueline Hughes, Gene Feder, Helen Cramer

**Affiliations:** ^1^ Department of Public Health and Primary CareUniversity of Cambridge School of Clinical Medicine, and Cambridge Biomedical Research CentreCambridgeUK; ^2^ Centre for Academic Primary CareSchool of Social and Community MedicineUniversity of BristolBristolUK; ^3^ Barts NIHR Biomedical Research UnitQueen Mary University of LondonLondon Chest HospitalLondonUK; ^4^ Department of Emergency MedicineJames Paget University HospitalGorleston‐on‐SeaNorfolk; ^5^ Department of MedicineUniversity of East AngliaNorwichUK; ^6^ Department of Epidemiology and Public HealthClinical Epidemiology GroupUniversity College LondonLondonUK; ^7^ School of Healthcare SciencesCardiff Metropolitan University

**Keywords:** advanced practice, non‐ST‐elevation myocardial infarction, nursing roles, specialist nurses, variation in care

## Abstract

**Background:**

Studies have shown variation in care for patients with non‐ST elevation myocardial infarction (NSTEMI), including in the roles of specialist and advanced practice nurses in diagnosis, treatment and coordination of care.

**Aim:**

The aim of this study was to describe the roles and responsibilities of specialist and advanced practice nurses in providing care for patients with NSTEMI.

**Methods:**

Secondary analysis of observational field notes and interviews from an ethnographic study of variation in care for NSTEMI patients in 10 UK hospitals conducted 2011–2012. Data were thematically analysed to identify key concepts and themes related to the roles of specialist nurses.

**Results:**

Seven of 10 hospitals had roles for specialist nurses in NSTEMI care. The major theme related to high demand and the complexity of patients and organizations (‘Aligning the planets’). In this theme, nurses contributed to improving services or compensating for deficiencies (‘Making the system work versus making up for the system’). Data collection for audit could take precedence over time with patients (‘Paying worship to the paper’). Nurses expressed a sense of ownership of cardiovascular patients that drove their desire to provide quality of care (‘They are our patients’).

## 1 Introduction

Myocardial infarction (MI) is defined as myocardial cell death caused by prolonged ischaemia and designated according to whether or not there is development of ST segment elevation in two or more contiguous leads on electrocardiogram (ECG): ST elevation MI (STEMI) or non‐ST elevation myocardial infarction (NSTEMI) (Thygesen et al., 2012). NSTEMI is more common than STEMI in older people, but it is complicated by greater uncertainty in diagnosis and management because of frequent atypical symptoms, lack of diagnostic ECG changes and patients’ age and comorbidities (Yeh et al., 2010; Zaman et al., 2014). Specialist and advanced practice nurses (APNs) have had varying roles in the care of patients with MI, including nurse‐initiated thrombolysis for STEMI prior to the wide‐spread adoption of primary percutaneous coronary intervention (PPCI). The role and scope of responsibilities of specialist nurses in diagnosing and managing NSTEMI patients has been less well‐defined. Few studies have evaluated the broader role of specialist chest pain or acute coronary syndrome (ACS) nurses and a survey of 192 UK emergency departments (EDs) in 2006 reported that specialist ‘chest pain’ or thrombolysis nurses were employed in 72% of EDs with chest pain units and 48% of hospitals overall (Cross, Howe, & Goodacre, 2007; Dunckley et al., 2006, 2007; Hamilton et al., 2008; Johnson, Goodacre, Tod, & Read, 2009; McLean, Phillips, Carruthers, & Fox, 2010; Smallwood, 2009; Tierney et al., 2013). The aim of our analysis was to describe the roles and responsibilities of specialist and advanced practice nurses in providing care for patients with NSTEMI in 10 hospitals in England and Wales.

## 2 Methods

Our data came from a large ethnographic study of processes that facilitated good quality care for patients with NSTEMI. Eight hospitals in England and Wales were purposively selected on the basis of variation (four in the top and four in the bottom tertiles) in 30‐day case mix adjusted mortality in the Myocardial Infarction National Audit Project (MINAP). Hospitals were also selected for variation in teaching status, geography, coronary intervention and patient volume. Field work methods were piloted in two additional hospitals and data from all 10 hospitals were used in the analyses. Hospitals were not aware of where they were on the spectrum of 30‐day cardiovascular mortality. Eleven hospitals were approached for participation and one declined to take part. All of the research team were blinded to hospital mortality data until after analysis of the data. Initial permission for interviews and observation was obtained from the cardiology clinical lead at each hospital and individual written consent was obtained prior to observation and interviews of staff and patients. Ethical approval was obtained from an NHS medical research and ethics committee (ref: 10/H0107/75).

Each hospital was visited by an experienced research associate who undertook an intensive 2‐week observation period between June 2011 and August 2012. During this period, the researcher observed activities, followed NSTEMI patients through their hospital course, reviewed medical records and interviewed patients, families, clinicians and managers. Patients and families were also interviewed 30 days after discharge. Observation and interviews took place in emergency departments, medical assessment units, coronary care units (CCU), catheter laboratories, cardiac and general medical wards and focused on the processes of admission, diagnosis, treatment and discharge. Selection of staff participants maximized variation in roles and patient participants were purposively selected for diversity of age, gender and clinical factors. Patients were introduced to the research associate by staff, who had ascertained if they were willing to talk about their experiences. Patients were visited more than once during the course of their hospitalization. Observational data were collected in detailed field notes and verbal data were audio‐recorded with permission and transcribed verbatim. A total of 732 hours of observation was conducted. Social desirability bias was minimized through comparison of observation and accounts of care and utilizing the perspectives of multiple staff and patients. Table 1 shows the number of patients and staff observed and interviewed and the total hours spent in observation in the 10 hospitals and Table 2 provides information about each hospital.

**Table 1:** Observations and interviews

Patients observed	68
Patients interviewed	53
Staff^a^ observed	199
Staff^a^ interviewed	142
Total hours on site in the 10 hospitals	732

a Staff included consultant physicians and physicians at various levels of training in cardiology and emergency medicine, nurses and nurse‐managers of wards/units and departments within the hospitals, specialist and advanced practice nurses, non‐clinical managers and administrative staff.

**Table 2:** Characteristics of hospitals in which ethnographic study was carried out

	Hospital 1	Hospital 2	Hospital 3	Hospital 4	Hospital 5	Hospital 6	Hospital 7	Hospital 8	Hospital 9	Hospital 10
Teaching status	Teaching (tertiary)	Non‐teaching	Teaching (tertiary)	Non‐teaching	Teaching (tertiary)	Non‐teaching	Teaching (tertiary)	Non‐teaching	Teaching (tertiary)	Non‐teaching
Volume of cardiac admissions 2008^a^	Medium	Medium	High	Low	High	Low	Low	High	High	Low
Type of MI patients	STEMI & NSTEMI	NSTEMI only	STEMI & NSTEMI	NSTEMI only	STEMI & NSTEMI	NSTEMI only	STEMI & NSTEMI	NSTEMI only	STEMI & NSTEMI	NSTEMI only
Primary PCI?	Yes	No	Yes	No	Yes	No	Yes	No	Yes	No
Type of intervention available	Angiogram PCI, CABG	None	Angiogram PCI, CABG	Angiogram PCI	Angiogram PCI	Angiogram PCI	Angiogram PCI, CABG	Angiogram	Angiogram PCI, CABG	Angiogram PCI
Specialist or Advanced Nursing Roles	ACS nurse (1)	ACS nurse (1)	ACS nurse (1)	Thrombolysis nurses (2)	No ACS nurses; Cardiac matron (1), senior sisters (2), junior sisters (4)	Chest pain nurses (5)	ACS nurses (4) (not as ED link but for inter‐hospital transfers)	ACS nurses (5)	No ACS nurses	No ACS nurses

a Low < 249, medium = 250‐499, high > 499.

ACS, acute coronary syndromes; CABG, coronary artery bypass graft surgery; ED, emergency department; MI, myocardial infarction; NSTEMI, non ST elevation myocardial infarction; PCI, percutaneous coronary intervention; STEMI, ST elevation myocardial infarction.

In the initial analysis of the data, it was evident that nurses were integral to the care of patients with NSTEMI, but their roles and responsibilities varied across the ten hospitals. The secondary analysis reported in this paper focused on specialist nurses and APNs who had responsibilities in identifying and managing patients with NSTEMI and who worked across different departments in the organization. The research team searched for relevant field notes and transcripts of interviews with or about specialist nurses. Four multi‐professional researchers then read and discussed the interviews and notes, focusing on nurses’ roles, contributions to processes of care and their perspectives on their work and care for patients with NSTEMI. A constant comparison approach (Strauss & Corbin, 1998) was used to identify similarities and differences in the data. Emerging themes and examples were discussed with other team members at one face‐to‐face meeting and refined via email and telephone exchanges. Themes and illustrative quotations and observations from field notes were organized using a spread sheet and shared electronically. All authors have reviewed and contributed to the final analysis and paper.

## 3 Results

Seven of the 10 hospitals had specialist nurses or APNs in key roles for patients with NSTEMI and in two other hospitals the senior nurse managers and cardiac rehabilitation nurses provided some similar activities. Analysis revealed extensive and diverse clinical and administrative responsibilities. The researcher observed myriad roles undertaken by specialist nurses in some hospitals and multiple job titles. The examples below are field notes from three of the hospitals:

Acute coronary syndrome (ACS) nurses – seem to do lots of other tasks too – arranging things, doing pre‐operative clinics for electives, stress echocardiograms, chest pain clinic, MINAP data. High demand, [barriers encountered] in getting things done…

‘Chest pain’ nurses [work] 7 days 7am – 7pm. Roles are different: booking in pts for pre‐elective checks, auditing the MINAP data and entering data; some teaching of electrocardiograms (ECGs) and other things

Cardiac specialist nurses: do troponin lists [i.e. review list of patients with elevated troponin levels], bed management for possible NSTEMIs. Also begin cardiac rehabilitation, conduct post MI clinic, pre‐angiography clinics, get called to review patients in the Medical Assessment Unit (MAU) and emergency department (ED).

From observations the roles of ACS nurses were varied and included activities to bridge care across departments and between inpatient and outpatient services. The major theme identified in the data related to the challenges of delivering timely and appropriate care to patients in complex systems with pressures from high service demand, treatment targets and professional and organizational boundaries (‘Aligning the planets’). In this complex system, a sub‐theme identified ACS nurses’ contribution to improving services or conversely trying to compensate for deficiencies in services (‘Making the system work versus making up for the system’). Another sub‐theme highlighted that data collection for quality improvement and audit could take precedence over time with patients (‘Paying worship to the paper’). Finally, ACS nurses expressed a sense of ownership of cardiac patients that drove their desire to provide quality of care despite obstacles (‘They are our patients’).

### 3.1 Aligning the planets

This encompassing theme reflected the complexity of organizing and providing care for patients with NSTEMI in hospitals under pressure: from initial diagnostic uncertainty to the difficulties of coping with transferred patients with NSTEMI (both urgent and elective cases) needing diagnostic angiography and possible PCI. Accommodating transferred patients when the receiving hospital had limited bed capacity and a full schedule in the cardiac catheterization laboratory was an organizational challenge requiring flexibility and coordination. The difficulty of coordinating care and ensuring that everything was in place was likened to a rare astronomical event where several planets are (from the earth's perspective) in the same region of the sky (Baird, 2013):

…and in order for it to work it was almost like aligning the planets so everything had to align. You had to have the beds, you had to have the cath lab space and you had to have the consultants and the nursing staff to proceed with that. [Advanced practice nurse]

In an organization, emergency departments and cardiology services were not always well aligned in policies, practice and communication and professional boundaries could interfere with best practice. Emergency department clinicians were often criticized by cardiology specialists for not investigating chest pain properly, resulting in unnecessary admissions. Nurses often found themselves in liaison positions between departments:

There is inevitably conflict between cardiology and A&E and I don't think there's ever not going to be, we are in the middle and we can see it from both sides so yeah I suppose we are kind of [a mediator or link]. [Cardiac specialist nurse]

One of the two hospitals without ACS nurses had previously had thrombolysis nurses, but the change to ACS nurse roles was not sustained and relationships between A & E and cardiology services suffered:

It used to be [better] links with A & E when thrombolysis was done in the department…because you had the [thrombolysis] nurses…when [primary]PCI came in their role became changed to an ACS nurse…the ultimate of the experienced nurses left…and the post was never…er, [the hospital has] just reabsorbed the funds from that post. [Consultant cardiologist]

Similarly, in the same hospital tensions between A & E and cardiology unit nurses were observed over appropriateness of transfers to the coronary care unit (CCU). The A & E nurses reported feeling caught in the middle between the A & E physicians and the CCU nurses who queried the appropriateness of the transfer. A senior A & E nurse thought that an ACS nurse could serve as a link between CCU and A & E staff and decrease the tension.

#### 3.1.1 Making the system work versus making up for the system

The diagnostic uncertainty and high demand for beds and services could often lead to systems that did not function well to find and treat patients. Observations in some hospitals indicated that ACS nurses were often employed to make up for system insufficiencies, although there were examples of nurses reforming the system to be more effective. In one example, the ACS nurses previously had spent most of their time on the telephone arranging transfers for NSTEMI patients needing angiography and intervention. The system did not differentiate between urgent and elective cases, with patients being transferred without triage or consideration of need:

[Transferred patients] were all kept on the same list and they came in order…our transfers and urgent work didn't seem to take priority. [Advanced practice nurse]

The ACS nurses worked with their hospital and transferring hospitals to make needed changes to the system: they were able to ‘ring‐fence’ or reserve beds for NSTEMI patients and they collaborated with the information technology service to create a confidential electronic referral form for elective patients. This allowed them to prioritize patients and importantly to spend time with patients rather than on transfer coordination. Making the system work also involved communication and support to transferring hospitals:

One of the things that having the electronic form does for us is that it gives us as nurse practitioners more time to spend with the patients than on the phone…

As a team we go to the [transferring hospitals] and [put on]teaching sessions on…how to refer…It builds networks…and people are not afraid to phone us and talk to us [if in doubt about a patient]. [Advanced practice nurse]

Some examples were more difficult to differentiate between whether ACS nurses were improving the system or covering for its deficiencies, but it was obvious that these nurses expedited patient treatment, management and discharge through advanced skills in assessment, diagnosis and risk stratification:

For us it's a simple thing that really makes the difference, which is the ACS nurses. … Sometimes they influence the general medical because … we can't see the patient at the outset. They will go quickly and see the patients and they are very experienced, they are very good, they will politely talk to the admitting team, to point out you know what needs to be done in terms of appropriate therapy for ACS patients, because some of the juniors aren't completely familiar…. so they do make a difference and obviously they alert us to the cases which are high risk and we see them quickly. So that's very, very good system I think, it's a very good system. [Cardiologist]

…we run a nurse assessment unit so we review cardiac patients on there and assess whether they're low risk or ACS patients… Well basically it is to prevent admission, aiding discharge and giving an early diagnosis of heart disease if at all possible, those patients that we don't think have an ACS but we think that there's a potential that their symptoms are cardiac in nature we best get them as an outpatient so we can try and catch them before it gets more serious, or maybe an ACS… [Cardiac specialist nurse]

In hospitals without ACS nurses, A & E staff often struggled to obtain cardiology support and cardiologists reported being stretched too thinly across the wards and catheterization laboratories to respond to A & E. In one hospital, the situation with cardiology was contrasted with the stroke service, which employed specialist stroke nurses:

And often if you try and get hold of a cardiologist, for instance, yesterday we were just ringing, getting given various bleep numbers and I rang CCU and they said oh you could try this bleep or you could try that bleep, … everyone works very much in silos, even within the hospital… [In patients with stroke] the stroke nurses made a huge difference. I mean I was really grateful that the stroke nurse suddenly came down yesterday and helped with the stroke patient… [ACS nurses] would be a fantastic role… it would make a huge difference. [Senior nurse, A & E]

ACS nurses were also involved in outpatient services such as rapid access chest pain clinics, which worked with varying degrees of success to decrease admissions or expedite discharge:

…[what] we are looking for is a middle ground … develop a service that means that okay the troponin is negative but I still think clinically this is cardiac chest pain and we have a service whereby you can come back in 24 hours’ time for a day case angiogram. And that's what we don't quite achieve at the moment. …we are looking at different routes … they could pull up at hospital and even in A&E we could say actually we don't need to admit you but come back tomorrow, present yourself at this desk and you will be seen by a consultant. So just much slicker, quicker routes to getting patients to the right place where they don't linger in hospital beds…. [ACS nurse]

Given the pressure for beds in most hospitals, patients admitted for possible NSTEMI but not considered urgent could be placed almost anywhere in the hospital. Patients whose subsequent troponin was elevated were not necessarily identified as potential NSTEMI by staff on the ward, so finding the patients with elevated troponin for assessment then became a major task for the ACS nurses:

…on a Monday we might come back to 25 abnormal troponins…we may end up with 5 MIs because there's a whole host of explanations [for elevated troponin]…now to some hospitals that's a mad way of doing it…because you end up reviewing case notes of patients that are not cardiac, but we've tried it every other way, we've tried to rely on the ward to refer when they get a cardiac patient with a raised troponin but they don't…’

‘And it is frustrating…I may find that nobody else has noticed that they have a raised troponin and ECG changes…So you do find those patients…but there's only one way to do it and that's physically go and get them… [Cardiac specialist nurse]

Some of the ACS or chest pain nurses were also involved in initiating cardiac rehabilitation, although across the hospitals cardiac rehabilitation was often poorly resourced. Staff noted that funding and support for rehabilitation services ebbed and flowed, but the service was the ‘poor man of cardiology’ and NSTEMI patients were not prioritised. Having a target for rehabilitation was seen as a potential driver for increasing resources for rehabilitation:

I think if there was some kind of [rehab] target then that would make a big difference. Because we are audited … and we have targets to achieve. [Chest pain nurse]

#### 3.1.2 Paying worship to the paper

The MINAP database provides important information about the outcomes of care for patients with MI and is a rich source of data for audit and research. However, the data for MINAP need to be collected from patient records, a time‐consuming task that often falls to nurses. Other quality improvement initiatives in the UK incentivise hospitals to document that evidence‐based care is being provided to patients, including those with MI. The demands of data collection and documentation of care could overwhelm other aspects of the specialist nurses’ role:

…on busy days I pay worship to the paper that I spend not enough time with the patient…I was far better at giving rehab…listening to patients and being there for them when I didn't have all of this to do…I might go and see a 50 year old guy and only have half an hour to spend with him because I spent 2 hours filling in 3 MINAP forms…and that's frustrating and that's why I think you get a bit demoralised with it really… [ACS nurse]

So it's got slightly better since we organised with [chest pain nurses] to do [MINAP data]…but they do get sidetracked more onto the paperwork side than the clinical side. [Consultant cardiologist]

Data collection forms can never capture all of the decision‐making and nuances of care, which could prove frustrating:

[documenting that a very ill patient was not on secondary prevention]….we're not trying to prolong his life, we're just trying to keep his remaining days comfortable, but MINAP doesn't ask that. … you can put not indicated or not applicable but it doesn't actually tell you anywhere what the reason is… [Cardiac nurse]

### 3.2 They are our patients

A sense of ‘ownership’ and responsibility for care of NSTEMI patients underlay the key processes of care with many ACS and other nurses expressing strong feelings of NSTEMI patients as ‘our patients’ and wanting to ensure that they were identified and provided with the best care:

…sometimes my [troponin] list can be down here and [the potential NSTEMI patients] can be all over the place, surgery, burns, elderly care, gastro, respiratory, they can be everywhere and orthopaedics and as cardiac nurses we'll go and visit every single one of those patients on that list. [Cardiac specialist nurse]

This sense of ownership could also involve circumventing the roles of managers coordinating patient placement:

It is very personal to us…because they are our patients…we see the cardiology patients, we get to know them and we want them cared for under our care. [Chest pain nurse]

if there is a patient that we are really concerned about and we think desperately needs a coronary care bed, we will get involved and we will ring coronary care ourselves and we will discuss it with coronary care… [Cardiac specialist nurse]

Yeah, we don't tell anybody, we just do it [move patients to get cardiac patients in cardiac beds]. I don't want to totally exclude the [bed] management, some of them don't acknowledge that we do this role. [Chest pain nurse]

Nurses’ concern for cardiac patients meant that they tried to ensure that cardiac patients received the care that they believed necessary. The nurse leading the rehabilitation service in one hospital confessed to following up patients with unstable angina post‐discharge by telephone despite this not being a service that was commissioned. Other nurses also found that talking to patients and understanding their individual situations led them to be less judgemental and to appreciate the reasons some patients persisted in unhealthy risk behaviours:

… I have at times gone in and read someone's notes and thought oh bloody hell what an idiot, you know, I can't believe what does it take [to change behaviour like smoking or diet]. He's got young kids and you go in, go in there feeling a little bit hmm and …then they start to talk to you and you realise their life is so [difficult]… [Cardiac nurse]

## 4 Discussion

This analysis has documented the versatility and varied roles and responsibilities of specialist cardiac and advanced practice nurses involved in the care of patients with NSTEMI. The variation in nursing roles and responsibilities for patients with NSTEMI highlights that these roles have developed in complex systems largely in response to service needs and preferences and skills of nurses and cardiologists. This is in contrast to nationally defined protocols such as nurse‐initiated thrombolysis in STEMI. For seven of the 10 hospitals studied, these nurses were central to ensuring patients with NSTEMI were identified, transferred as appropriate, managed, educated, often followed up as out‐patients and that care was documented for audit. These are broadly consistent with the roles identified in a previous review of nurses’ contribution to NSTEMI care: patient assessment, triage, coordination of care and education (Tierney et al., 2013), although the roles, responsibilities and activities identified in this study were more extensive. The other three hospitals studied did not have these specific roles although in two of the other hospitals senior cardiac nurse managers and rehabilitation nurses undertook some similar activities. Although the data were not collected to explore ACS nurse roles, staff in hospitals without these posts did note a need for the role especially related to liaison between departments and support for providers. Cardiology services often found themselves too stretched to be able to respond quickly to A & E requests for assessment and departmental relationships were often tense. Although patient interviews were scrutinised, no data related specifically to ACS nurses was found in these.

We also had no data on the effect of ACS nurse roles on patient outcomes, but other studies have documented the impact of nursing roles on outcomes (David, Britting, & Dalton, 2015; Johnson et al., 2009; Mehta et al., 2006; Smallwood, 2009). Patients with STEMI, NSTEMI and heart failure in the USA admitted to coronary care by a team that included a cardiac advanced practice nurse (CAPN) had 30‐day re‐hospitalization rates 50% lower than those admitted by a team without the CAPN. Although the 185 patients (78% NSTEMI) were not randomised, there were no significant differences by demographics, clinical characteristics or interventions between the groups. The better outcomes were attributed to the continuity provided by the CAPN, relationships built with multi‐professional teams in acute and community services, expertise developed and the teaching and family support provided by the CAPN (David et al., 2015). Other studies have found greater NSTEMI patient satisfaction with information when a specialist nurse was involved and greater adherence to guideline‐based care (Johnson et al., 2009; Mehta et al., 2006; Smallwood, 2009). Many of the ACS nurses were involved in audits of evidence‐based care and were in a position to report on adherence and remind providers of recommended treatment. Others commented on ACS nurses’ influence on care provided especially when less experienced medical or nursing staff were involved. Their roles in ‘finding’ ACS patients admitted throughout the hospital and in moving patients to cardiac wards could also affect the evidence‐based care provided.

Although the ACS nurses may have contributed to improving services at times, their roles often seemed to be trying to make up for system deficiencies or intervening where differing priorities and perspectives, or professional boundaries interfered with delivering care. Frustrations were frequently expressed regarding targets, funding and resources for services and the challenges of organizing and delivering care in a high‐pressure healthcare system. Observation by the research associate documented the intricacies of delivering care and the multiple problems that often occurred and needed to be resolved in complex organizations.

The strength of the study lies in its multiple perspectives in interviews and longitudinal observations providing an in‐depth view of processes of care and interaction among providers, patients and systems in 10 varied hospitals. The analysis is limited by being a secondary analysis of data collected for a study of variations in care across the 10 hospitals and the lack of opportunity to question and focus on ACS nurse roles. Effort was made to include a wide variety of providers in different roles and to minimise bias through multiple interviews and observations, but important persons and factors may have been missed. The findings are not linked to patient outcomes from the hospitals and may not be transferable to other settings. Nonetheless, the analysis does provide an extensive view of the roles and activities of ACS nurses in providing care for NSTEMI patients.

## 5 Conclusion

In contrast to national or hospital protocol‐driven roles of nurses in management of STEMI (primarily initiation of thrombolysis), roles related to NSTEMI patients have developed from the bottom up based on needs in hospitals and to some extent the expertise and preferences of nurses and cardiologists. Identified themes illustrated the specialist nurses’ roles in coordinating and facilitating appropriate care, their roles in changing or compensating for dysfunctional systems and the frustrations inherent in delivering care for heterogeneous and often complicated patients in complex systems. Greater understanding of these roles and their effectiveness in improving patient care and outcomes would be beneficial.

## Funding

This study was funded through a Research for Patient Benefit grant from the National Institute of Health Research, UK.

## Conflict of interest

No conflict of interest has been declared by the authors.

## Author contributions

All authors have agreed on the final version and meet at least one of the following criteria [recommended by the ICMJE (http://www.icmje.org/recommendations/)]:

- substantial contributions to conception and design, acquisition of data, or analysis and interpretation of data;
- drafting the article or revising it critically for important intellectual content.
